# Primary malignant solitary fibrous tumor of the neck in an HIV-positive patient: a case report

**DOI:** 10.3389/fonc.2026.1880093

**Published:** 2026-08-19

**Authors:** Jing Liu, Siying Zhu, Cheng-xi Wang, Yuejuan Zhao

**Affiliations:** 1 Department of Medical Imaging, Yunnan Provincial Hospital of Infectious Disease, Kunming, Yunnan, China; 2 Department of Ultrasonography, Yunnan Cancer Hospital, The Third Affiliated Hospital of Kunming Medical University, , Kunming, Yunnan, China

**Keywords:** bone tumor, HIV infection/AIDS, imaging diagnosis, malignant solitary fibrous tumor, solitary fibrous tumor

## Abstract

We report a rare case of malignant solitary fibrous tumor (MSFT) in the cervical region presenting with aggressive bone destruction and lung metastasis in a 54-year-old HIV-infected male. The patient, who was on long-term antiretroviral therapy (EFV + 3TC+TDF), presented with a 5-month history of a progressively enlarging mass in the posterior neck (approx. 10.0 cm x 8.0 cm x 4.0 cm) accompanied by radiating pain in the right upper limb. Initial imaging revealed an osteolytic, destructive lesion involving the C3-C5 vertebrae and multiple solid pulmonary nodules, suspicious for malignancy. Preliminary biopsy and immunohistochemistry initially suggested a mesenchymal malignant tumor, possibly a BCOR-altered sarcoma. Following a multidisciplinary team (MDT) consultation and according to the 2024 CSCO guidelines for bone and soft tissue tumors, the patient received two cycles of neoadjuvant chemotherapy (Epirubicin 30mg D1-D3 and Ifosfamide 2g D1-D5). Subsequent surgical resection was performed, and definitive histopathological and immunohistochemical analysis of the postoperative specimen confirmed MSFT (STAT6+, CD34+, P53 mutant type ~85%, and Ki-67 ~60%). This case emphasizes the diagnostic challenge of differentiating rare spindle cell sarcomas in HIV-infected patients; high-grade MSFT can mimic other aggressive sarcomas, and comprehensive postoperative pathological evaluation, especially STAT6 staining, is essential for definitive diagnosis and tailoring oncological management.

## Introduction

Human immunodeficiency virus (HIV) infection is strongly associated with immune dysregulation and an increased susceptibility to various neoplasms. While AIDS-defining cancers like Kaposi sarcoma are well-documented, the occurrence of rare mesenchymal tumors, such as solitary fibrous tumor (SFT), remains exceptionally sparse in this population (1). SFT is a rare spindle cell neoplasm characterized by the NAB2-STAT6 gene fusion (2, 3). Its malignant counterpart, malignant solitary fibrous tumor (MSFT), is even rarer, exhibiting aggressive clinical behavior, extensive bone destruction, and a high potential for distant metastasis (4, 5).

The clinical and radiological presentation of MSFT in the cervical region can be highly non-specific, often mimicking other high-grade sarcomas or infectious processes, which increases diagnostic complexity in immunocompromised patients (6, 7). Notably, certain subtypes of rare sarcomas, such as BCOR-altered sarcoma, share overlapping histopathological features with MSFT, potentially leading to initial diagnostic pitfalls (8). Given the aggressive nature of these tumors, distinguishing MSFT from its mimics is crucial for determining the appropriate neoadjuvant therapy and surgical strategy (9).

Imaging plays a vital role in identifying vertebral destruction and staging, yet definitive diagnosis relies heavily on comprehensive immunohistochemical (IHC) profiling and molecular analysis (7). This report details a rare case of a 54-year-old HIV-infected male with a rapidly progressing cervical MSFT that was initially suspected to be a BCOR-altered sarcoma. By describing this diagnostic evolution, we emphasize the importance of integrated pathological evaluation and the utility of markers such as STAT6 and Ki67 in the precise management of rare sarcomas in the context of HIV infection.

## Case report

**Figure 1:**
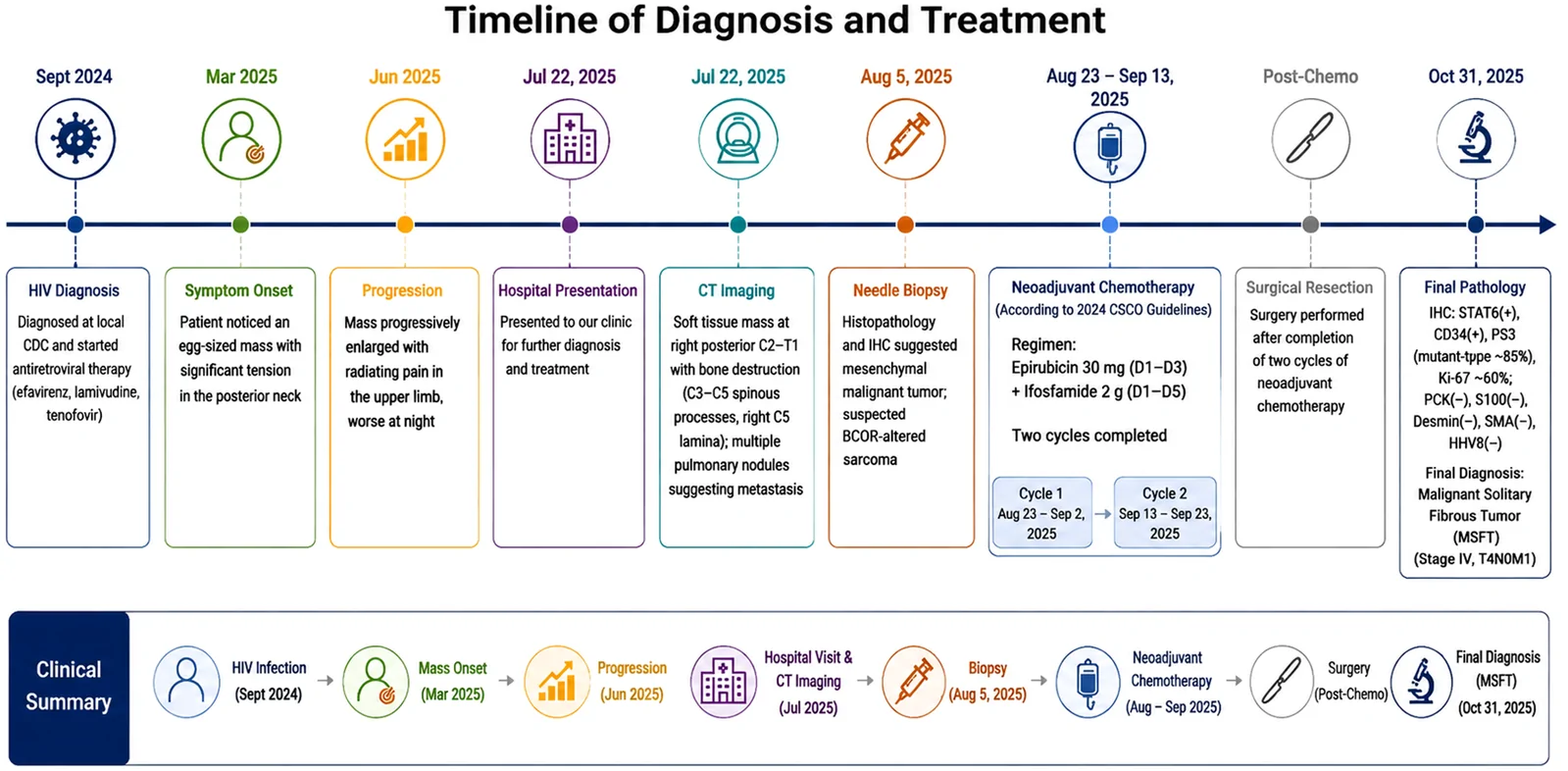
Timeline of diagnosis and treatment. This schematic illustration was generated using ChatGPT (OpenAI, GPT-5.5-mini, https://chat.openai.com/) based on the authors’ original clinical data. All information presented in the figure was reviewed and verified by the authors.

A 54-year-old man had a documented history of human immunodeficiency virus (HIV) infection. He was diagnosed with HIV infection at the local Center for Disease Control and Prevention (CDC) in September 2024 and subsequently received regular antiretroviral therapy consisting of efavirenz, lamivudine, and tenofovir. In March 2025, he first noticed an egg-sized mass in the posterior neck with increased local tension but without significant pain or functional impairment; therefore, no further medical evaluation or treatment was sought at that time. The lesion gradually enlarged thereafter. Approximately three months later, the patient developed radiating pain in the right upper extremity, which progressively worsened and became particularly severe at night, accompanied by restricted movement of the right middle, ring, and little fingers. Owing to the continuous enlargement of the mass and the development of neurological symptoms, he presented to our hospital on July 22, 2025, for further evaluation and treatment (**Figure 1**). Physical examination revealed a firm, fixed, high-tension mass measuring approximately 10.0 cm × 8.0 cm × 4.0 cm in the posterior neck, without fluctuation. The right middle, ring, and little fingers were maintained in a flexed position with limited extension.

**Figure 2:**
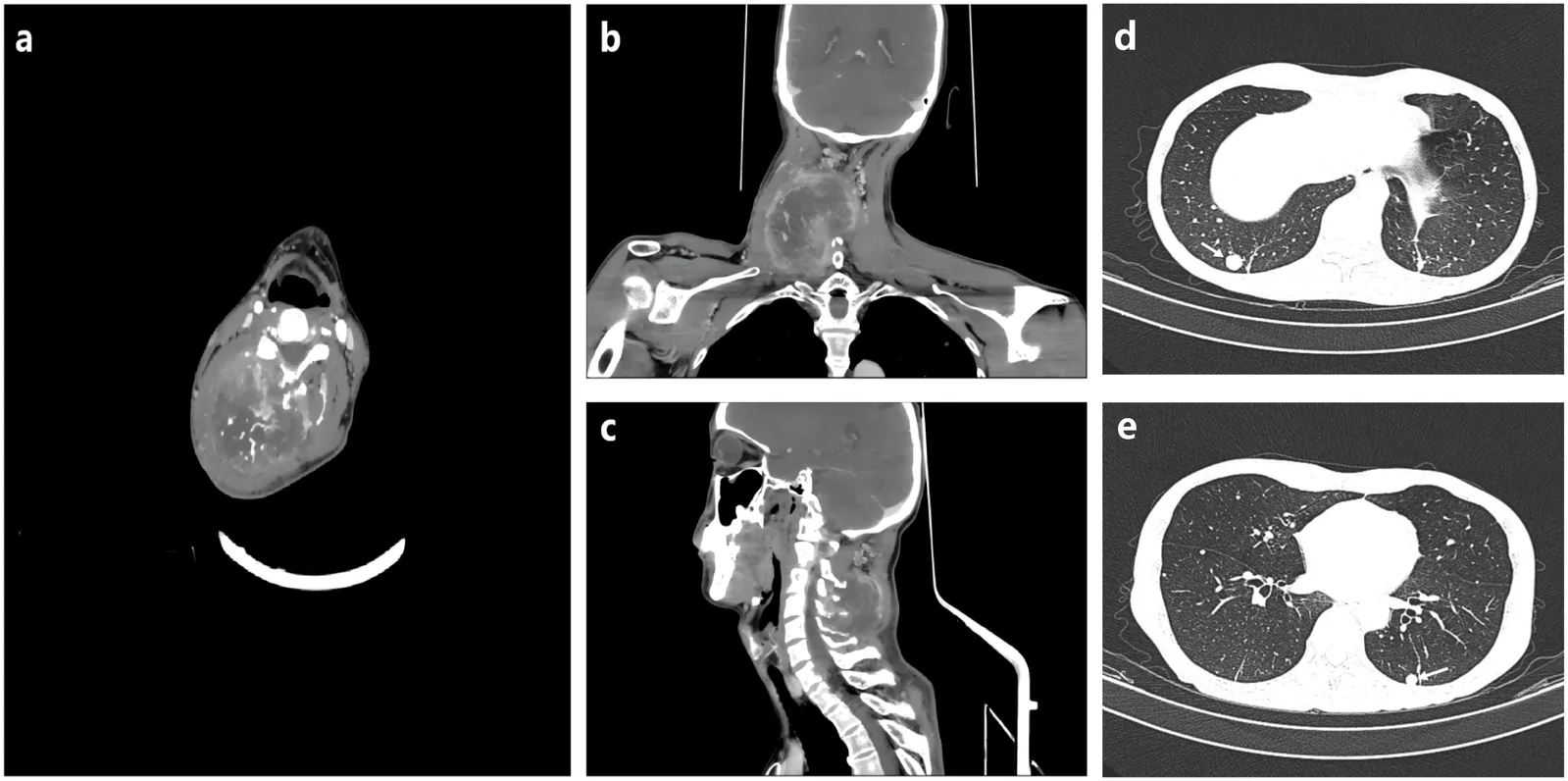
Contrast-enhanced CT images of the cervical malignant solitary fibrous tumor. **(a)** Axial contrast-enhanced CT image demonstrating a large heterogeneous soft tissue mass located in the right posterior cervical region, with prominent internal enhancement and areas of necrotic change. **(b)** Coronal reconstructed CT image showing the superior–inferior extent of the lesion involving the posterior aspect of the cervical spine. **(c)** Sagittal reconstructed CT image revealing an expansive posterior cervical mass extending from approximately C2 to T1, accompanied by destruction of the C3–C5 spinous processes and the right C5 lamina, suggestive of aggressive malignant behavior. **(d, e)** Axial chest CT images showing metastatic nodules in both lungs (white arrows) with additional scattered bilateral pulmonary micronodules, confirming bilateral pulmonary metastatic involvement.

Computed tomography (CT) imaging revealed a soft tissue mass at the right posterior aspect of the C2-T1 vertebrae, with associated bone destruction of the C3-C5 spinous processes and the right lamina of C5, highly suggestive of a malignant tumor. Additionally, multiple solid pulmonary nodules were identified, indicating potential metastasis (**Figure 2**). Physical examination upon admission revealed a fixed, high-tension mass in the posterior neck, measuring approximately 10.0 cm × 8.0 cm × 4.0 cm, with no fluctuance or neck stiffness noted.

On August 5, 2025, a needle biopsy of the neck mass was performed. Initial histopathological and immunohistochemical (IHC) analysis suggested a mesenchymal malignant tumor. Due to the limited biopsy sample, the preliminary diagnosis was suspected to be a BCOR-altered sarcoma. Following a multidisciplinary team (MDT) consultation and according to the 2024 CSCO guidelines for bone and soft tissue tumors, the patient initiated neoadjuvant chemotherapy on August 23, 2025. The regimen consisted of two cycles of Epirubicin (30 mg, D1–D3) and Ifosfamide (2 ag, D1–D5).

**Figure 3:**
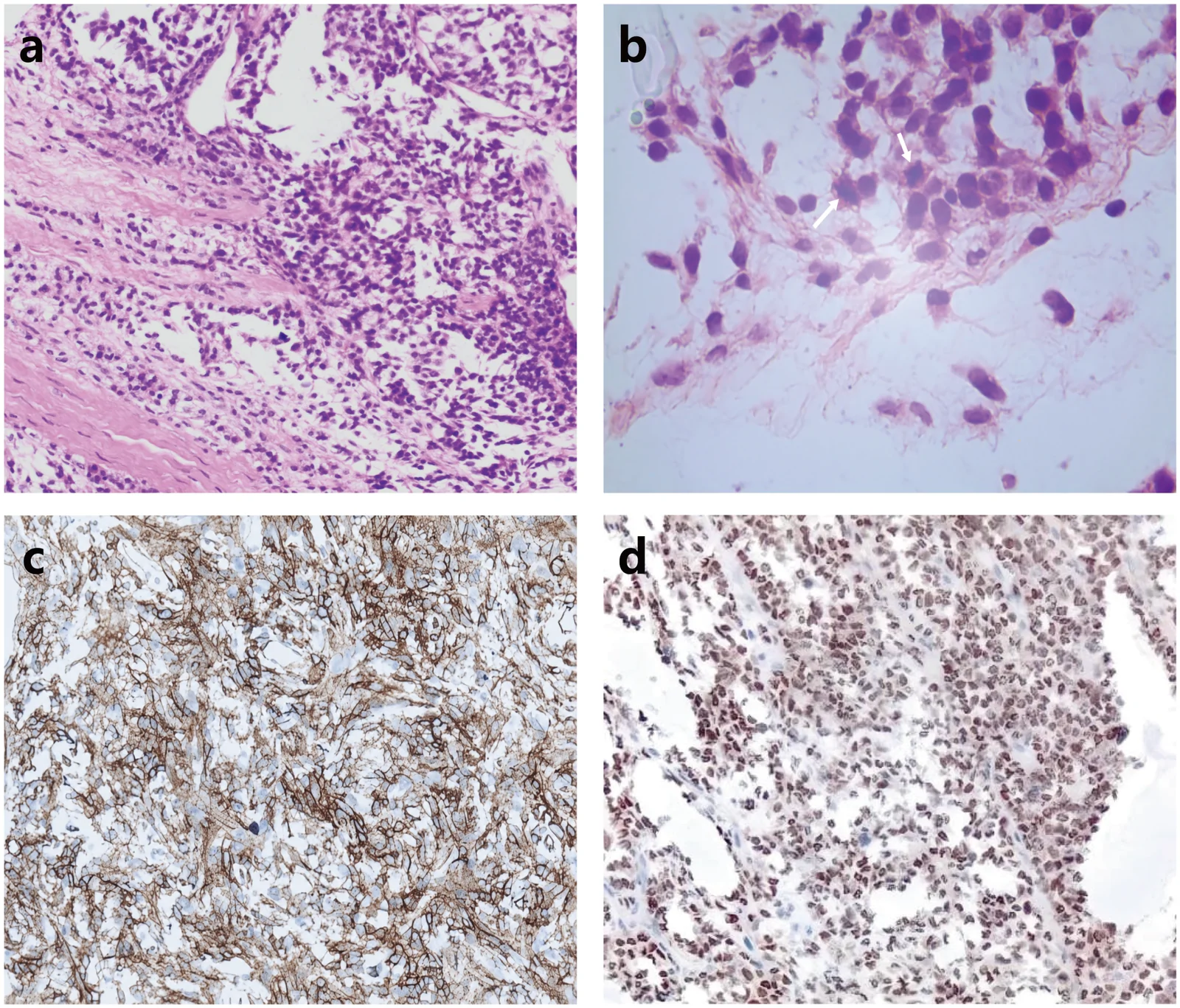
Histopathological and immunohistochemical findings of the primary cervical malignant solitary fibrous tumor (MSFT). **(a)** Hematoxylin and eosin (H&E) staining at ×200 magnification: The tumor is hypercellular with variable collagenous stroma; tumor necrosis and characteristic branching thin-walled “staghorn-like” blood vessels are observed. **(b)** H&E staining at ×400 magnification: Cellular-rich areas are predominantly composed of round to short spindle-shaped neoplastic cells, belonging to the spindle cell tumor spectrum, with marked nuclear atypia. Mitoses are frequently identified (approximately 10 mitoses/10 HPF), and typical mitotic figures are marked by white arrows. **(c)** Immunohistochemical staining for CD34: Diffuse positive staining is detected in tumor cells, accompanied by positive staining of supportive vascular endothelial cells. **(d)** Immunohistochemical staining for STAT6: Diffuse strong nuclear positivity is exhibited in tumor cells.

Following the completion of two chemotherapy cycles, the patient underwent surgical resection. The definitive postoperative pathological evaluation, completed on October 31, 2025, corrected the diagnosis. Histopathological examination of the resected specimen revealed a hypercellular spindle-cell neoplasm consisting of short spindle to ovoid tumor cells arranged in irregular fascicles within a variably collagenous stroma. Characteristic branching thin-walled staghorn-like blood vessels were observed. Marked nuclear atypia, increased cellularity, frequent mitotic activity (~10 mitoses/10 HPF), and tumor necrosis were identified. Since the specimen was submitted as multiple fragmented tissue pieces, invasive growth could not be reliably evaluated. Immunohistochemical assays showed diffuse strong nuclear STAT6 staining (3+) in 95%–100% of tumor cells, along with positive CD34 expression (**Figure 3**). Overexpression of mutant p53 protein was detected in approximately 85% of neoplastic cells, and the Ki-67 proliferation index was markedly elevated (around 60%).Tumor cells were negative for PCK, S100, Desmin, SMA, HMB45, HHV8, CD31, and CK5/6, while Fli-1 and ERG staining was limited to vascular endothelial structures rather than tumor cells.

Although the initial needle biopsy suggested the possibility of BCOR-altered sarcoma because of limited sampling and overlapping immunophenotypic features, evaluation of the complete surgical specimen demonstrated morphological and immunophenotypic findings supporting solitary fibrous tumor. Considering the presence of characteristic staghorn vasculature, diffuse STAT6 nuclear expression, high mitotic activity, extensive necrosis, and synchronous pulmonary metastases, the final diagnosis was established as malignant solitary fibrous tumor (MSFT) (Stage IV, T4N0M1). Molecular confirmation by NGS was not available and remains a limitation of this report.

## Conclusion

The clinical course of this case highlights the diagnostic complexity and the aggressive nature of malignant solitary fibrous tumor (MSFT) presenting as primary cervical bone destruction. SFT is generally considered a borderline or low-grade mesenchymal neoplasm; however, the malignant variant (MSFT) can exhibit high-grade features, including rapid growth, extensive osteolysis, and early systemic dissemination (5). In the context of HIV infection, the rarity of MSFT, combined with its non-specific radiological appearance, creates a significant diagnostic challenge. As seen in this patient, the extensive vertebral destruction and pulmonary nodules could easily be misidentified as more common HIV-associated malignancies or infectious processes.

A key learning point from this case is the “diagnostic evolution” from an initial suspicion of BCOR-altered sarcoma to a definitive diagnosis of MSFT. This shift underscores the inherent limitations of needle biopsy and small-tissue sampling in the evaluation of rare spindle cell sarcomas (10). Because MSFT shares significant morphological overlap with other high-grade mesenchymal tumors—such as BCOR-altered sarcoma or undifferentiated pleomorphic sarcoma—a definitive diagnosis frequently necessitates the examination of the complete surgical specimen (11, 12).Strong and diffuse nuclear expression of STAT6 serves as a highly sensitive and specific marker for distinguishing MSFT from its histological mimics, while CD34 expression provides supportive but non-specific evidence. A high Ki-67 proliferation index reflects increased tumor aggressiveness rather than serving as a primary diagnostic criterion (11, 13).

From a pathological perspective, SFTs are variably cellular tumors composed of ovoid to spindled cells exhibiting pattern less growth or a storiform pattern against a variably collagenous background stroma containing thin-walled large branching, “staghorn”-shaped (HPC-like) blood vessels (14). Medium sized blood vessels with perivascular fibrosis are also commonly seen (15, 16). Several clinicopathological features have been associated with malignant behavior, including large tumor size, increased cellularity, nuclear pleomorphism, elevated mitotic activity, tumor necrosis, and infiltrative growth patterns (17, 18). In the present case, the initial core needle biopsy suggested the possibility of BCOR-altered sarcoma due to limited sampling and overlapping spindle-cell morphology. However, evaluation of the complete surgical specimen demonstrated characteristic histological features of SFT, including branching thin-walled (“staghorn-like”) vessels, increased cellularity, and nuclear atypia. Immunohistochemically, although SFTs commonly express CD34, CD99, and BCL-2, diffuse nuclear STAT6 expression is currently regarded as the most sensitive and specific surrogate marker for the NAB2–STAT6 gene fusion, which is the molecular hallmark of SFT (19, 20).In this patient, the complete postoperative specimen exhibited diffuse and strong nuclear STAT6 expression (approximately 95–100%). The combination of this robust positivity with CD34 expression not only strongly supported the diagnosis of SFT but also served as the critical evidence to clearly distinguish it from other spindle-cell sarcomas, particularly the initially suspected BCOR-altered sarcoma.

A limitation of the present case is the absence of molecular testing. Although the diagnosis was supported by characteristic histopathological and immunohistochemical findings, comprehensive molecular analysis, including assessment of NAB2–STAT6 fusion, was not performed. Such testing may provide additional diagnostic confirmation and further improve the understanding of the molecular characteristics underlying this rare tumor.

Furthermore, the development of this tumor during effective antiretroviral therapy (ART) is a notable observation. It suggests a “decoupling” between viral suppression and the emergence of non-AIDS-defining mesenchymal malignancies (1). In HIV-infected individuals, persistent genomic instability and alterations in the immune microenvironment may provide a permissive landscape for rare sarcomas, regardless of the CD4+ T-cell count or viral load. This emphasizes that clinical vigilance for malignancy must be maintained in the HIV population, even when the underlying infection is well-controlled (21, 22).

In summary, for HIV-infected patients presenting with isolated, destructive bone lesions, MSFT should be a key consideration in the differential diagnosis. Early recognition of invasive features on imaging, prompt multidisciplinary team (MDT) evaluation, and the pursuit of definitive histopathological evidence—specifically STAT6 staining—are essential. Adhering to these diagnostic and therapeutic strategies is critical for ensuring precise oncological intervention and improving the prognosis for patients with such aggressive and rare neoplasms.

## Data Availability

The original contributions presented in the study are included in the article/supplementary material. Further inquiries can be directed to the corresponding authors.
